# Spontaneous Bacterial Peritonitis Caused by *Listeria monocytogenes* Associated with Ascitic Fluid Lymphocytosis: A Case Report and Review of Current Empiric Therapy

**DOI:** 10.1155/2013/832457

**Published:** 2013-09-15

**Authors:** Todd Yecies, Sanae Inagami

**Affiliations:** ^1^University of Pittsburgh School of Medicine, 4601 Bayard Street, Pittsburgh, PA 15213, USA; ^2^University of Pittsburgh and VA Pittsburgh Healthcare System (VAPHS), Pittsburgh, PA 15213, USA

## Abstract

Spontaneous bacterial peritonitis (SBP) is a potentially deadly complication of ascites. We describe a case of SBP caused by *Listeria monocytogenes* in a patient with alcoholic cirrhosis. This was associated with the unusual finding of ascitic fluid lymphocytosis, which previously had only been associated with tuberculoid or malignant ascites. Given increasing rates of cefotaxime-resistant SBP alongside the possibility of Listeriosis, the use of cefotaxime as first-line therapy in SBP should be reevaluated.

## 1. Introduction

Spontaneous bacterial peritonitis is the most common life-threatening infectious complication of cirrhosis, affecting between 10 and 30% of cirrhotic patients hospitalized for ascites [1]. Hospital mortality rates range from 10 to 30%, with the development of progressive renal impairment being the strongest predictor of mortality [2].

SBP is thought to result from increased bacterial translocation across gastrointestinal mucosa combined with diminished host immunity in cirrhotic patients [2]. Enteric gram-negative bacteria are the most common cause of SBP, with *E. coli *and *Klebsiella* responsible for more than half of SBP cases [3]; however there is a recent evidence for increasing prevalence of SBP caused by gram-positive cocci [4]. In addition, cases of SBP caused by *Candida*, anaerobes, and *Listeria *have been reported [3].

Intravenous cefotaxime is considered standard of care for empiric therapy for SBP after it was demonstrated superior to ampicillin-amikacin [5]. However, recent studies suggest that cefotaxime resistance is increasingly common [4]. Other studies show that empiric amoxicillin-clavulanic acid or ciprofloxacin may be equally effective in select patients [6, 7]. Appropriate selection of empiric antibiotics is essential as absolute mortality rates in patients not covered by the initial antibiotic therapy were 20% higher than in patients with appropriate coverage [8].

Cefotaxime-resistant bacteria that can cause SBP include extended-spectrum beta-lactamase (ESBL) producing gram-negative rods, *Enterococci*, anaerobes, and *Listeria *[9]. Fewer than 5 cases of *Listeria *SBP have been reported in the United States.

## 2. Case Report

This patient is a 62-year-old Caucasian male with a history of alcoholic cirrhosis and ascites who presented with one month of increasing abdominal distention and discomfort. His distention had been worsening since his last paracentesis one-month prior. He was afebrile and otherwise asymptomatic on presentation. Prior to this hospitalization, he had undergone 8 therapeutic large-volume paracenteses over the past year. His past medical history was significant for stable grade-IV small lymphocytic lymphoma, an unrepaired umbilical hernia, chronic hyponatremia, and chronic obstructive pulmonary disease. His medications included furosemide, spironolactone, omeprazole, thiamine, and citalopram. 

His vital signs on admission showed a temperature of 37.1°C, pulse of 102, blood pressure of 110/71, and respiratory rate of 18. On exam he had significant abdominal distention, shifting dullness, and a positive fluid wave consistent with ascites. His abdomen was diffusely tender to palpation with rebound and voluntary guarding. His umbilical hernia was easily reducible, but the overlying skin was warm, indurated, erythematous, and tender. The patient was admitted for therapeutic paracentesis and treatment of cellulitis. 

Labs at presentation demonstrated WBC of 8.9 with 81% neutrophils, Hgb of 12, INR of 1.6, Na of 125, K 4.9, Cr of 0.6, albumin 2.9, T. bili 2.8, AST 87, ALT 32, and AP of 117. His MELD score was 16. Blood cultures were drawn. Bedsides paracentesis was performed which collected 3250cc of cloudy fluid which showed 2000 RBCs and 6000 WBCs, of which 86% were neutrophils, and 2000 RBCs. Ascitic fluid gram stain was negative. He was started on 1 g IV cefotaxime Q8h and 1.5 g/kg IV albumin following the paracentesis for treatment of SBP. 

On day 2 of admission, the patient developed a fever to 38.6°C and his abdomen remained diffusely tender to deep palpation despite improved distention. His umbilical cellulitis improved daily on IV cefotaxime with decreasing erythema, local tenderness, and induration.

On day 3 he continued to be febrile to 38.9°C. On day 4, cefotaxime was discontinued and ampicillin/sulbactam was initiated after ascitic fluid cultures grew *Listeria monocytogenes*. Blood cultures drawn on admission remained negative. Repeat paracentesis obtained on day 4 also grew *Listeria *and demonstrated persistent elevation of ascitic WBCs to 4280 with a lymphocytic predominance (48% lymphocytes, 43% neutrophils). The patient responded clinically to the change of antibiotics with improved abdominal tenderness and discomfort. 

On day 8 the patient underwent a third paracentesis, which grew no organisms and demonstrated a decline in total WBCs to 2660 and increased lymphocytic predominance (57% lymphocytes, 33% neutrophils). After completing 5 days of treatment with ampicillin/sulbactam, the patient was discharged with a 9-day course of amoxicillin/clavulanic acid. A subsequent paracentesis demonstrated complete resolution of his SBP. 

## 3. Discussion

*Listeria monocytogenes* is a facultative anaerobic zoonotic gram-positive rod. Transmission occurs fecal-orally from contaminated food or animal products. Up to 5% of the general population may be asymptomatically colonized by *Listeria*. Infection with *Listeria* most commonly occurs in neonates, the elderly, and those with suppressed cell mediated immunity. 

*Listeria* most commonly causes bacteremia and meningitis, though it can also cause gastroenteritis, endocarditis, and SBP [10]. *Listeria* is a rare cause of SBP with fewer than 5 documented cases in the United States. Listerial SBP can be fatal with reported mortality rates of up to 30% [11]. Cirrhosis and hematologic malignancies are known risk factors for listeriosis, and a high index of suspicion for listeriosis must be maintained in alcoholics or patients with liver disease [12]. In this patient the presence of cirrhosis with comorbid small lymphocytic lymphoma may have depressed his cell-mediated immunity and increased his susceptibility to *Listeria*. 

The delay in starting appropriate antibiotic therapy significantly increases mortality in SBP cases [9]. While antibiotic coverage for rare organisms should not compromise coverage of common ones, this case report demonstrates the limitation of current standard empiric therapy for SBP. Two recent studies have documented increasing rates of cefotaxime-resistant SBP, with reported treatment failure rates of 43% and 41% [8, 9]. When considering empiric therapy for SBP, the chosen antibiotic should cover gram-positive cocci, an increased range of drug resistant bacteria, and the rare but susceptible organism like *Listeria. *Cefotaxime and other third-generation cephalosporins increasingly fail to meet these criteria. Piperacillin/tazobactam and meropenem are antibiotics with documented efficacy in SBP that treat *Listeria* and also improve coverage of gram-positive cocci and ESBL-producing gram-negative rods [8, 9]. Given these facts, it is reasonable to initiate broad-spectrum coverage with piperacillin/tazobactam or meropenem for empiric treatment of SBP. The relative efficacies and complications of empiric therapy for SBP with these agents should be studied.

An unexpected finding in this case is the late presence of lymphocyte predominance in the ascitic fluid. A previous case of monobacterial nonneutrocytic bacterascites caused by *Listeria* also demonstrated lymphocytic predominance in ascitic fluid [13]. Meningitis caused by *Listeria* is associated with lymphocyte predominance in CSF fluid [10], and it is possible that a similar mechanism leading to lymphocytic predominance may occur with listerial SBP. Ascitic fluid lymphocytosis has previously only been reported with tuberculoid peritonitis or peritoneal carcinomatosis [14].

## 4. Conclusion

*Listeria monocytogenes* is a rare but potentially fatal cause of spontaneous bacterial peritonitis. Lymphocytic predominance in ascitic fluid may be suggestive of *Listeria* infection. Current empiric therapy regimens for SBP should be reevaluated in light of high rates of cefotaxime resistance, and broad-spectrum empiric therapy with piperacillin/tazobactam or meropenem should be considered in patients not responding to empiric treatment.
